# Protracted Oxidative Alterations in the Mechanism of Hematopoietic Acute Radiation Syndrome

**DOI:** 10.3390/antiox4010134

**Published:** 2015-02-27

**Authors:** Nikolai V. Gorbunov, Pushpa Sharma

**Affiliations:** Department of Anesthesiology, Uniformed Services University of the Health Sciences, Bethesda, MD 20814, USA

**Keywords:** ionizing radiation, hematopoietic acute radiation syndrome, electrophilic stress, radiation-induced oxidation, nitration, carbonylation, non-targeted epigenetic and clastogenic effects, homeostatic tissue barriers, vascular injury, intracranial hemorrhage

## Abstract

The biological effects of high-dose total body ionizing irradiation [(thereafter, irradiation (IR)] are attributed to primary oxidative breakage of biomolecule targets, mitotic, apoptotic and necrotic cell death in the dose-limiting tissues, clastogenic and epigenetic effects, and cascades of functional and reactive responses leading to radiation sickness defined as the acute radiation syndrome (ARS). The range of remaining and protracted injuries at any given radiation dose as well as the dynamics of post-IR alterations is tissue-specific. Therefore, functional integrity of the homeostatic tissue barriers may decline gradually within weeks in the post-IR period culminating with sepsis and failure of organs and systems. Multiple organ failure (MOF) leading to moribundity is a common sequela of the hemotapoietic form of ARS (hARS). Onset of MOF in hARS can be presented as “two-hit phenomenon” where the “first hit” is the underlying consequences of the IR-induced radiolysis in cells and biofluids, non-septic inflammation, metabolic up-regulation of pro-oxidative metabolic reactions, suppression of the radiosensitive hematopoietic and lymphoid tissues and the damage to gut mucosa and vascular endothelium. While the “second hit” derives from bacterial translocation and spread of the bacterial pathogens and inflammagens through the vascular system leading to septic inflammatory, metabolic responses and a cascade of redox pro-oxidative and adaptive reactions. This sequence of events can create a ground for development of prolonged metabolic, inflammatory, oxidative, nitrative, and carbonyl, electrophilic stress in crucial tissues and thus exacerbate the hARS outcomes. With this perspective, the redox mechanisms, which can mediate the IR-induced protracted oxidative post-translational modification of proteins, oxidation of lipids and carbohydrates and their countermeasures in hARS are subjects of the current review. Potential role of ubiquitous, radioresistant mesenchymal stromal cells in the protracted responses to IR and IR-related septicemia is also discussed.

## 1. Introduction

Numerous reports indicate that any given dose of ionizing irradiation (IR) can affect at certain degree all cells, biological fluids and entire organ systems. However, there are limiting doses, which can trigger acute radiation sickness due to impairment of radio-sensitive systems and tissues, such as hematopoietic, lymphoid, gut mucosa, and vascular endothelium, development of acute reactive responses, and loss of systemic homeostatic control [1,2,3,4,5,6,7,8,9,10,11,12].

Hematopoietic form of the acute radiation syndrome (hARS) due to total body irradiation (TBI) is characterized by a depletion of the peripheral white blood cells, suppression of clonogenic potential and impairment of bone marrow, lymphoid tissue, mucosal epithelium and vascular endothelium that can ultimately lead to late-stage attrition of immune, intestinal, and vascular barriers accompanied by: (i) hypercytokinemia and protracted non-septic inflammatory, neurogenic, metabolic, oxidative and electrophilic stress; (ii) coagulopathy; (iii) enteropathy; (iv) fluid loss, electrolyte imbalance, interstitial edema; and (v) *Enterobacteriaceae* bacteremia and sepsis [1,2,3,4,5,9,10,11,12,13,14,15,16]. All together these alterations are considered to be major factors of morbidity and moribundity in hARS.

hARS is distinguishable from gastro-intestinal (GI), or cardio-pulmonary, or neurovascular ARSs by the characteristic symptoms and lethality. Thus, compared to hARS, the symptoms of the last forms of ARS appear at higher IR doses and within a shorter time-lag, where lethal outcomes are inevitable [1,3,4,5,9]. In laboratory mice hARS can be produced by total-body irradiation (TBI) with X-rays and gamma-rays [1,2,9]. The doses required for hARS vary from 5.0 Gy (gray) through 9.7 Gy (for the median lethal doses over 30-days observation period, LD_50/30_; note Gy is the SI unit of absorbed radiation) depending on the animal strain [1,2,9,10,17,18]. Nonetheless, it is well documented that there are common patho-physiological patterns in sequelae of the radiation sicknesses in animal models and human victims of radiation accidents and radiotherapy patients [3,9,12,19,20,21].

**Figure 1:**
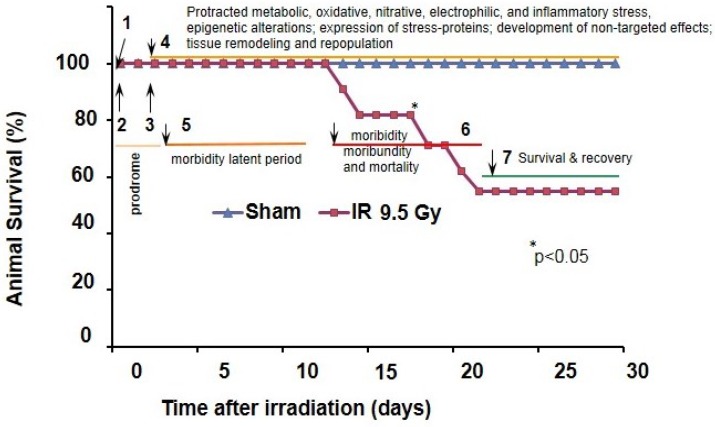
Kaplan–Meier survival plot and post-irradiation (post-IR) events in mouse model of the hematopoietic acute radiation syndrome (hARS). 1—Radiolysis due to pulse-irradiation and associated formation of (i) electrophilic and nucleophilic species; (ii) reactive oxygen and nitrogen species (ROS and RNS); (iii) electrophil-derived danger-associated molecular patterns (DAMPs), pro-inflammatory oxysterols, and clastogenic plasma factors in the target-cells and fluids. Time-lag is minutes; 2—Induction of cell and organ system responses to the targeted and non-targeted effects including redox-stress due to disruption of mitochondrial redox circuitry in the photon-targeted mitochondria; electrophilic stress; epigenetic changes. Time-lag is hours; 3—Direct cytocidal response (time lag is from hours through two to three days, end of prodrome); Development of clonogenic suppression, acute phase response, non-septic inflammation, lymphopenia, neutropenia, immunosuppression (time lag is days); 4—Protracted oxidative, nitrative, electrophilic and proteotoxic stress; development of clastogenic, metabolic and epigenetic responses; tissue remodeling and repopulation. Time lag for the reactive response is days; 5—Morbidity latent period: regressive hematological changes, development of coagulopathy and anemia, impairment of tissue barriers. Time-lag is 1–1.5 week; 6—Enteric bacteria breach the gut barriers; development of bacteremia, interstitial hemorrhage, moribundity and mortality (time-lag is 1–1.5 week); 7—Recovery during post-survival period (time-lag is days). The survival plot is adapted with modifications from: Kiang *et al.*, 2014 [23]. Experimental conditions: hARS was induced by exposure of B6D2F_1_/J mice to 9.5 Gy whole-body bilateral ^60^Co gamma-photon radiation, delivered at a dose rate of 0.4 Gy/min (LD_50/30_).

Development of the hARS pathophysiological conditions in mice occurs in a sequential mode within 1.5–3 weeks post-irradiation period with a peak of moribundity between the 12th and 18th day post-irradiation over 30-days observation period. A schematic summary of the hARS-related events is shown in Figure 1, which represents a Kaplan–Maier survival plot for hARS (LD_50/30_ for B6D2F1/J mice) and a sequence of the pathophysiological onsets in the post-IR period.

Overall, this sequence can be presented as follows: the targeted and non-targeted radiation effects lead to gradual decline in the lymphatic, hematopoietic and immune functions, induction of hypercytokinemia and non-septic inflammation, delayed onset microvascular dysfunction, and decrease in integrity of mucosal barrier that facilitate bacterial translocation; while the secondary impact can occur when bacterial pathogens and inflammagens spread through the vascular network resulting in septicemia. At this stage, the secondary, septic inflammatory response can exacerbate the systemic vascular dysfunction, and accelerate failure of organs and systems [22]. Evidently, these complex pathophysiological processes in hARS are frequently culminate in moribundity Figure 1 and [1,10,19,21].

Recent reports indicate that improvement of animal survival from hARS and dose reductions can be produced by administration of antibiotics, immunomodulators, growth factors (e.g., G-CSF, bFGF), and growth hormone secretagogues in post-IR period [15,17,19,21,23,24,25]. Compelling benefits of these approaches confirm that onset of delayed outcomes of acute IR is associated with non-targeted, epigenetic effects, dysregulation of systems interactions, suppression of clonogenic potentials, widespread dysfunction of tissue barriers, and bacteremia. Numerous molecular/cellular mechanisms driving these pathophysiological processes have been investigated, determined and proposed [26,27,28,29]. Among them is a plethora of uncontrolled multistep oxidative events and redox stress triggered during irradiation. Thus, the first step of the IR-induced (direct) oxidative damage occurs upon radiolysis of cellular constituents and biofluids due to fluence of ionizing particles through molecular targets followed by a massive formation of a wide range of electrophiles (e.g., free radicals, ROS, reactive carbonyl species (RCS), electrophilic nitroalkenes), electrophil-derived danger-associated molecular patterns (DAMPs), pro-inflammatory oxysterols, and the pro-inflammatory and atherogenic Michael adducts that are recognizable by Toll-like receptors (TLRs)/the pattern-recognition receptors (the PRR), as well as activation of epigenetic mechanisms [27,30,31,32,33,34,35,36,37,38]. In these events, according to a pecking order for free radical electron (hydrogen atom) transfer reactions, a depletion of free-radical quenchers, e.g., low molecular weight antioxidants, such as ascorbate, glutathione, and tocopherols, which constitute redox buffers in tissues and fluids, would occur in the first order [39,40]. Therefore, treatment with antiradical/antioxidant agents (e.g., amifostine and gamma-tocotrienol) has been a one of long-term strategies for radioprotection and suppression of systemic responses such as non-septic inflammation [14,20,41]. Secondly, the radiolysis effects on the cell redox metabolic machinery (e.g., endoplasmic reticulum (ER)-mitochondrial network, constituitive nitric oxide synthase (NOSI), peroxisome, *etc.*) and the systemic responses to the radiation-produced blood plasma DAMPs and inflammagens create a ground for development of the metabolic stress, non-septic inflammation, and the “functional redox stress” characterized by excessive metabolic production of ROS, the reactive nitrogen species (RNS) and reactive electrophils [6,7,20,27,33,42,43,44,45,46,47,48,49,50,51,52,53]. Ultimately, associated increase in the gap junction intercellular communication and release of these reactive products by the responsive cells can propagate non-targeted radiation effects [42]. Therefore, applications of modulators of: (i) mitochondrial redox function; (ii) the ER-stress response; (iii) antioxidant adaptive responses; (iv) renin-angiotensin system; (v) peroxisome proliferaton; (vi) activity of NADPH oxidase 4; and (vii) ROS/RNS-induced programmed cell death, are under recent consideration for mitigation of radiation-related effects [8,20,44,51,54,55,56]. Thirdly, the targeted and non-targeted epigenetic alterations, up-regulation of inflammatory mediators can lead to a long-term expression of pro-oxidant genes (such as inducible nitric oxide synthase, NOSII), which can further exacerbate the metabolic oxidative/electrophilic stress [51,57,58]. As a countermeasure,, development of new approaches using antioxidant gene therapy for dose-limiting tissues is also under consideration [43,59].

As shown in Figure 1 the next “wave” of the delayed oxidative stress in hARS would be expected upon septic inflammatory response to bacterial breach and appearance of bacterial inflammagenes in circulatory system. Altogether, these mechanisms can drive different stages of the protracted oxidative/electrophilic stress, which can self-propagate with diffusible reactive factors through the intercellular gap junctions and biofluid networks, and thus, can sustain delayed indirect radiation effects in the pathogenesis of hARS. Management of these IR-induced pathogenic factors remains to be a challenging task. In this light, up-regulation of the adaptive mechanisms of resistance to DAMPs and inflammagens with immunomodulators [such as the newly introduced Entolimod acting via Toll-like receptor 5; nuclear factor (NF-κB); activator protein 1 (AP-1); signal transducer and activator of transcription 3 (*i.e.*, STAT-3); and nuclear factor erythroid derived 2-related factor-2 (*i.e.*, Nrf2), a crucial regulator of the antioxidant/electrophile response element (ARE/EpRE) motif] is also considered to be a promising perspective countermeasure against hARS [60].

The presence and mechanisms of prolonged oxidative stress in the irradiated tissues is of a great recent interest [6,7,20,43,44,51]. This phenomenon has been documented in different tissues and systems, such as hematopoietic, lung, gut crypts, and microvasculature that sustain homeostatic barriers [20,43,49,51,59]. However the origin of cell which promote this oxidative stress in a long run, remains unclear.

Remarkably, recent *ex vivo* and *in vitro* experiments demonstrate that IR and/or bacterial inflammagens can prompt the radio-resistant lineages of mesenchymal stromal cells (MSCs) to pro-oxidant alterations [49,57,61]. In parallel to these pro-oxidant events, the “primed” cells are capable of activating a cascade of stress-adaptive responses and mechanisms of cell remodeling including autophagy/mitophagy [61,62]. Therefore, it seems that although these redox-proactive cells can cause oxidative effects in their milieu they are able to resist to “self-inflicted” oxidative damage [57,62]. Considering that MSCs are essential constituent of the ubiquitous stromal tissue and thus present in diverse tissue barriers, e.g., gut crypt units, bone marrow, lung, microvasculature, *etc.* [8,49,57,63,64,65], it would be reasonable to assume that MSC response to IR can contribute to protracted oxidative stress in hARS. IR-activated redox mechanisms and the role of ROS, RNS, and reactive products of oxidation/nitration of lipids, nucleic acids, and proteins (amino acids) in the targeted and non-targeted radiobiological effects including mechanisms of homeostatic regulation and impairment of tissue barriers in the mouse model of hARS are subjects of this minireview.

## 2. Radiation-Related Multistage Activation of Oxidative Reactions and Their Role in hARS

Radiation energy absorbed by tissues and fluids is dissipated by the radiolysis of water and biomolecules [30,31,32,33,34]. Thus, a pulse radiation impact to a single cell can *per se* produce instant severe damage to vital biomolecules and organelles that control transcriptional and translational events, cell metabolic homeostasis, communication, growth, differentiation, and death. The redox-reactive products resulted from the target impacts, *i.e.*, hydroxyl radical (HO*), nucleophilic hydril (H*) and hydrated electron (e^−^_aq_), and an array of biomolecule-derived carbon-, oxygen-, sulfur-, nitrogen-centered radicals (*i.e.*, RC*, RO*, RS*, and RN*), and labile iron [30,31,32,33,34]. A part of these “primary” radical species is subjected to subsequent quenching by antioxidants with the diffusion-dependent rate constants [32,33,34], while other enable to further propagate oxidative stress [47]. Indeed, according to the pecking order for electrophilils, the strongly electrophilic HO* has a capacity to oxidize organic molecules with the diffusion-dependent rate constants [30,66]; other “primary” free radicals and e^−^_aq_ recombine with oxygen to form numerous peroxides and superoxide anion radicals (O_2_*^−^) under aerobic conditions [31,33,39,47,48,67]. These “secondary recombinant” reactive oxygen species (ROS) can be either promptly utilized in the Haber–Weiss/Fenton-type reactions and, by these means activate the free-radical and radical-free metabolic pathways, or subjected to termination by antiradical/antioxidant redox mechanisms or intramolecular recombinations [27,33,39,66,68,69,70,71,72]. As an endpoint of these free free-radical reactions is a rise of non-radical electrophils in the targeted cells and biofluids that can either sustain or interfere with homeostatic response mechanisms. It seems that the “secondary” ROS could potentially amplify the “primary” oxidative stress; however, theoretical calculations indicate that the yields of products generated as a consequence of a primary ionization event and essential for “secondary” oxidative hit are lower by orders of magnitude than that produced by normal cellular metabolism [47]. Therefore, the concept of amplification of radiation-induced “primary” oxidative stress has been further refocused on shift of metabolic and pro-inflammatory redox pathways in irradiated cells [20,44,45,47,48,53].

Oxidation of biomolecules due to radiolysis and the Fenton mechanism has been extensively investigated over past decades. For a long time the radiobiological effects were associated with IR-produced clustered DNA lesions, *i.e.*, two or more individual lesions within one or two helical turns of the DNA that occur after passage of a single radiation track through a nucleus (Note mitochondrial DNA (mtDNA) are equally susceptible to IR-injury), [26]. Irradiation can produce clustered DNA cleavage through the direct impact of the ionizing photons on the DNA as well as through the indirect action of reactive chemical species formed near the DNA due to radiolysis and oxygenation. Indirect effects are attributed to oxidative damage by ROS, primarily by hydroxyl radicals generated in radiolysis and the Fenton reactions [26,27,66,73].

However, while the “direct” damage of DNA produced by photons occurs randomly in sugar and base moieties that leads to strand-breakage, release of free (unaltered) DNA bases; phosphates; and the formation of intermediate DNA free radicals and the TBA, *i.e.*, 2-thiobarbituric acid, reactive products; the hydroxyl radicals react with the bases of DNA rather than the sugars [66]. In these events, the main reaction is addition of the hydroxyl radical to the π-bonds of the bases. In the presence of oxygen, the resulting pyrimidine carbon-based radicals can be converted to the corresponding peroxyl radicals, as are sugar-based radicals. Hydroxyl radical adducts of purines can be further subjected to cleavage or underwent recombination. Ultimate intra-molecular recombination of the base-centered radicals can lead to oxidation of the base and sugar moieties and the oxidative DNA cleavage [26,27,32,33,34,66,67,73,74,75].

While the targeted DNA damage and epigenetic alterations are considered to be crucial mechanisms of IR-induced cell death and the radiation-induced mutagenesis and genomic instability, the emerging role of other type of biomolecules modified due to radiation exposure is of growing recent interest. Thus, alike to nucleic acids and nucleotides, the deleterious effects of IR on protein (peptides, amino acids), carbohydrates, and lipid polyunsaturated fatty acids (PUFA) can occur either due to their radiolysis followed by formation of RC*, ROO*, RO*, RS*, and RN*, or due to oxidation by ROS, primarily by hydroxyl radicals, resulted from radiation exposure and the Fenton reactions. This free radical oxidation rise an array of reactive electrophils, which can cause further post-translational modification proteins, activation of cell defense mechanisms, or detrimental effect on homeostatic responses [27,37,74,76]. Recently defined important electrophils produced due to irradiation and peroxidation are the protein, amino acid and lipid-derived carbonyls (reactive with 4-Dinitrophenylhydrazine (DNPH) to form hydrazones) [18,33,34,68,69,70,77,78]. Direct protein carbonylation, *i.e.*, post-translational modification resulting in the addition of reactive carbonyl groups to a proteins, occurs as a consequence of oxidation of lysine, arginine, histidine, proline, glutamate, and threonine residues, and fragmentation products of peptide bond cleavage reactions. Protein carbonyls are reported to be detectable a while after radiation exposure [27,33,78,79].

IR-induced oxidation of lipids generates a great deal of reactive intermediates, *i.e.*, reactive lipid species (RLS). Thus, IR-induced cleavage of PUFA or abstraction of a proton from PUFA by hydroxyl radical leads to addition of molecular oxygen to form PUFA-peroxides followed by their chemical degradation (Hock cleavage). These peroxidation reactions yield a plethora of PUFA-derived electrophilic (“soft”) carbonyls, which include by α,β-unsaturated aldehydes and ketones, such as 2-alkenals, 4-hydroxy-2-alkenals (4-HNE), 4-oxo-2-alkenals (4-ONE), acrolein, and malondialdehyde (MDA). [27,79]. The molecular construction containing α,β-unsaturated carbonyl conjugated with the diene displays efficient electron-withdrawing properties when reacting with nucleophiles, such as cysteine thiol, lysine, or histidine residues, via Michael mechanism, producing a variety of intra and intermlecular covalent adducts. These covalent modifications result in a free carbonyl attached to the protein that appears as “secondary” carbonylation [27,35,79]. In addition to carbonylation, the PUFA-derived aldehydes can react with the amine moiety of lysine residues to form the Schiff base adducts which can further undergo intraprotein recombinations [79]. Note, RLS such as MDA or 4-HNE can react with DNA and RNA (nuclear and mitochondrial) as well; in particular, with the guanin and adenin bases yielding etheno adducts (*i.e.*, ethenobases). The produced aberrations in the nucleic acids can lead to mis-transcriptions and thus to altered transcriptional products. Moreover, by building Schiff-bases with histones and other transcription regulatory proteins in the nucleus, these RLS can provoke clastogenic effects and promote epigenetic alterations.

Notably, PUFA derivatives with conjugated dienes are particularly susceptible to *in vivo* nitration with endogenously produced RNS yielding nitroalkenes, another group of electrophilic RLS. [35].

Biological effects of α,β-unsaturated aldehydes and ketones have been well addressed in recent reports and associated with multi diverse interference of these products with the cell protein machineries situated in cytosol and crucial organelles (e.g., mitochondria, ER, nuclei), and formation of immunogens, inflammagens, and DAMPs in the biofluids recognizable by PRRs on non-targeted cells [26,37,38,74,79,80,81]. Based on these observations, post-translational modification of proteins with PUFA-derived carbonyls, *i.e.*, RLS, have been also proposed as a novel signaling mechanism that modulates the cell redox-stress responses including those mediated by Nrf2, NF-κB, heat-shock proteins 70 and 90, heat-shock factor 1 (HSF-1), and APE/EpRE [35,70,74,75,79,80]. It should be noted that excessive generation of RLS implicated in the Michael Addition reactions can spread carbonyl (electrophilic) stress from the targeted cells to bystander cells via the gap junction network or the extracellular vesicle mechanisms thus producing non-targeted effects including mitochondrial dysfunction, ER-stress, disturbance of calcium homeostasis, and epigenetic and clastogenic dysregulations [74,79,82].

Overall, the “radiolysis-phase” has a short-term character but causes devastating systemic impacts. Thus, the IR-induced damage to proteins and lipids, post-translational modification of proteins, impairment of DNA, epigenetic alterations and formation of aberrant organelles generate an array of mediators of stress, danger and inflammation that interfere with cell communication systems and homeostatic control [16,20,33,40,41,44,51]. In conjunction with these events, activation of free radical reactions, formation of ROS, RNS, RCS, RLS, the Schiff and etheno adducts, the products of protein sulfhydryl oxidation as well as depletion of antioxidants are the major feature of radiolysis in tissues and fluids; and therefore, are considered to be biomarkers of oxidative stress in radiation exposure. To address adverse effects of this pro-oxidant shift the concept of antioxidant countermeasure against IR has been widely elaborated. To date, numerous antioxidants were proposed as litigators of the acute radiobiological effects [20,40,41,55,56,59]. So far, there is only one of them approved by the FDA for the radioprotection: this is amifostine (WR2721), an antiradical phosphorothioate [41].

Recent observations indicate that IR-induced radiolysis can affect crucial cellular constituents, skew calcium homeostasis, and thus can activate a cascade of metabolic responses leading to a prolonged oxidative stress propagating systemically [47,50,51]. The emerging evidence on this phenomenon include *in vitro*, *in vivo* and clinical data obtained from analysis of vasculature, lung, bone marrow, white blood cells, and gut mucosa [18,20,43,44,46,49,57,77,82]. There are several redox mechanisms, which are proposed to drive the metabolic oxidative stress. Among them are activation of: (i) constitutive and inducible nitric oxide synthases [40,57]; (ii) NADPH oxidase [44]; (iii) monoamine oxidase [41,42]; (iv) radiation-inducible microRNA miR-193a-3p [51]; (v) transient receptor potential (TRP) proteins [68]; (vi) peroxisomes [43]; and (vii) ER-mitochondrial axis [37,39,44]. Respectively, nitroxidative post-translational modification of proteins by nitric oxide (NO)—derived reactive nitrogen species has emerged as crucial factor of prolonged oxidative stress in hARS [42,57,68]. In this light of particular role is IR-induced disbalance of mitochondrial redox machinery, which is the major consumer of oxygen in aerobes as well as the major source of metabolically produced ROS in most cells [39,42,43,45]. This fact is especially important considering that the mitochondrial volume represents a substantial radiation-target, *i.e.*, 4%–30% cell-volume depending on the cell type [39,45]. Recent extensive investigations of radiation effects on mitochondria have defined that these organelles bear superior role in response to radiation hit by triggering (i) short-term and long-term metabolic responses (e.g., a decrease in oxidative phosphorylation); (ii) metabolic amplification of the “primary” ROS-yield from radiolysis; and (iii) the cytochrome C-dependent mechanism of programmed cell death [16,39,43,44,45,83,84]. Numerous *in vitro* and *in vivo* observations indicate that radiation effects in mitochondria have multifactorial character.

Firstly, mitochondrial DNA is very susceptible to radiation damage. In response to the formation of lesions irradiated mitochondria can up-regulate expression of specific mitochondrial genes that are related to cell survival; and they can also induce a compensatory increase in the mitochondrial DNA copy number, *i.e.*, “mitochondrial polyploidization” [53]. The reactive products of radiolysis such as ROS, RCS, RLS can produce post-translational modifications of mitochondrial proteins followed by functional alterations, e.g.,: (i) a prolonged dysregulation of the respiratory electron transport chain (affecting Complex I, Complex II, Complex III, and succinate/pyruvate-mediated respiratory capacity) with subsequent increase in ROS production; (ii) alterations in oxidative phosphorylation; (iii) remodeling of mitochondrial-ER network, activation of mitophagy, and change in the mitochondrial mass; (iv) increase in intra-organelle communications and [Ca^2+^]-mediated propagation of oxidative stress; (v) increase in permeability and swelling [6,47,50,52,53,61,68,81,84]. All together these reactions may constitute the “mitochondrial ROS-induced ROS” mechanism [85], and thus sustain the effect of prolonged oxidative stress in irradiated tissue [47,50,53,84]. Referring to the discussed above reports, γ-photon-targeted impacts on a mitochondrial-ER construct can cause impairment of mitochondrial respiratory redox complex, increase in membrane permeability of the organelles and subsequent transient release of mitochondrial/ER Ca^2+^, reversible depolarization of the mitochondrial membrane potential, and activation of Ca^2+^-mediated inter-mitochondrial communication for propagation of damage signal with a subsequent amplification of ROS yield, activation of the constitutive NOS, and formation of RNS, RLS, an array of reactive carbonyl derivatives. In-vivo assessment of these events in hARS is a challenging proposition, nevertheless, some recent reports have confirmed the phenomenon of protracted up-regulation of ROS and oxidative/nitrative post-translational modification of proteins in different tissues including those which exert barrier functions [20,43,44,49,51]. Thus, IR-associated the “metabolic” activation of ROS can further sustain and propagate the systemic electrophilic stress to form a variety non-targeted effects in tissues and organ systems.

**Figure 2:**
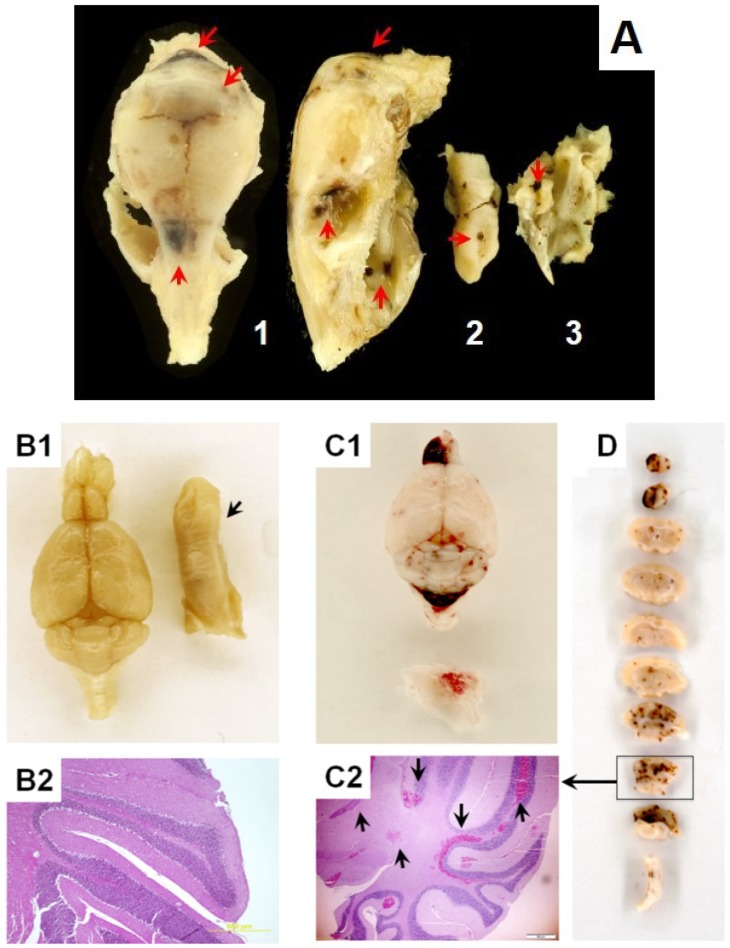
Development of hemorrhagic vasculopathy in irradiated B6D2F1/J mice at the mortality period. Gross pathology and histopathology assessment (hematoxylin and eosin staining, *i.e.*, H & E) of intracranial hemorrhage in moribund B6D2F1/J mice subjected to hARS. Panel **A**. (1) Images a mouse skull: dorsal plane (left) and lateral plan (right); (2) Image of tongue; (3) Image of a fragment of skull shown in (1). Brownish areas of extravasated blood are indicated with red arrows. Panels **B1** and **B2** are specimens from a sham animal. Tongue is indicated with a black arrow in **B1**. Panels **C1**, **C2**, and **D** are specimens from an irradiated animal. A fragment of cranium is indicated with a black arrow in **C1** where the presence of epidural hemorrhage is observable. Panels **B2** and **C2** display H & E-staining images of coronal sections through the cerebellar cortex. Panel **D** displays gross-image of coronal sections through entire brain. As shown in **C1** and **D** subdural and interstitial hemorrhage randomly occurred in different part of the brain; predominantly affecting cerebellum and olfactory. Experimental conditions: as indicated in the Figure 1.

Adverse effects of ionizing radiation on tissue barriers and loss of homeostatic control of organs and systems have been the major radiation concern since early ages of Radiation Medicine dated from the beginning of the 20th century [86]. In 1930’s John H. Lawrence and co-authors extensively investigated hARS in animals and reported the presence of *Enterobacteriaceae* bacteremia after a week following irradiation; they defined sepsis as a factor of the observed animal mortality [1]. Importance of bacterial breach of the tissue barriers and sepsis in radiation injury has been broadly documented in clinical observations and a variety of animal models over past decades [2,3,10,11,19,20,87]. Interestingly, it has been recently communicated that development of immunosuppression in irradiated animals can increase susceptibility to bacterial inflammagens and septicemia in orders of magnitude compared to control [10,87]. This phenomenon can suggest a crucial role of the secondary impact of the septic inflammation in vascularture and parenchymal tissues sensitized by irradiation. In conjunction with these events, one would expect another “wave” of oxidative stress driven by septic responses in the affected systems [22,88,89]. Moreover, the presence of bacterial inflammagens in the IR-sensitized microvasculature can lead to the generalized hemorrhagic Shwartzman-like reaction. Indeed, a widespread interstitial hemorrhage (in GI tract, lung, liver, and brain) due to microvascular rupture has been documented in moribund B6D2F1/J mice (Figure 2), but not in those animals that about recovering from hARS over the 30 days-post-IR period. The blood extravasation is shown to cause the hemoglobin-mediated oxidative stress, which can lead to shock and animal death; and therefore, can be considered as the ultimate mechanism of oxidative stress in hARS. The role of particular cell lineages in these vascular effects is under investigation [20].

Referring to the discussed above mechanisms of the radiation-related stress and biochemical alterations driven by ROS, RNS, and RCS, all of them can be implicated in different stages of hARS. Thus, the time lag for the cytocidal end-points in the radiation cell-target model depends on the cell biological characteristics, *i.e.*, efficacy of stress-response mechanisms and repair, cellular constituents, metabolic specificity, the kinetics of the target-cell population, and the physiology and construction of related tissue; that overall, determine the cell radiosensitivity [3,8,11,13,20,51,61,77]. As reported the time lag for the radiosensitive target-cells is 1–2 days [3,8,11,54,77,90] that is corroborated with onset for the hARS prodrome defined in Figure 1. This post-IR period is characterized by development of acute phase response, non-septic inflammation, lymphocytopenia, neutropenia, and suppression of clonogenic activity in the mucosa, bone marrow, and vascular endothelium [5,10,11,12,13,17,19,20]. Evidently, these alterations are leading factors that provoke the further bacterial breach of the gut mucosal and blood immune barriers, and induce sepsis. In the mouse model of hARS the appearance of moribund animals occurs after 10–12 days following IR; while the animal loss is continuing over 7–10 days [10,17,20,21,23,77,91]. Dramatic metabolic changes occur within this post-IR period that can include oxidative, nitrative, and carbonyl stress to sensitive tissues, e.g., the bone marrow and small intestine [18,20,24,44,49,77,78]. These assessments in the irradiated tissues can lead to establishment of a connection between protracted oxidative stress, the redox metabolome and functional genome that mediate redox homeostasis [92,93].

## 3. Radiation-Induced Oxidative Reactions and Related Alterations in Tissue Barriers

**Figure 3:**
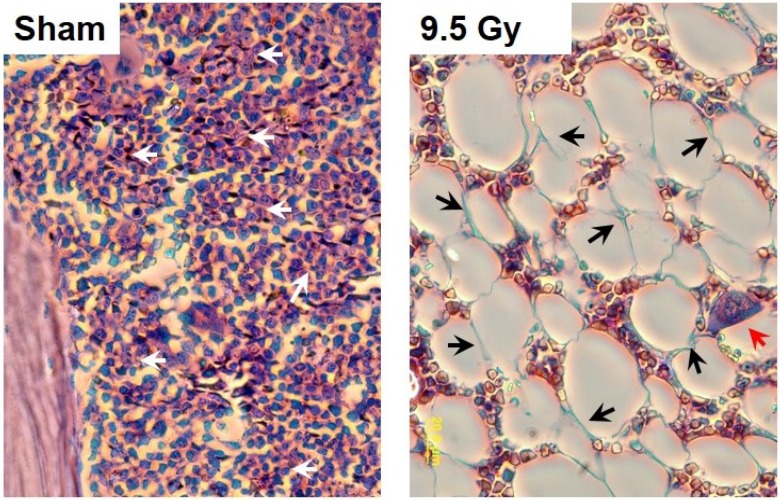
Radiation-induced depletion of bone marrow tissue with hematopoietic cells occurs in mice experienced hARS. Hematopoietic cells are indicated with white arrows in a “sham” specimen (left panel). Depletion with hematopoietic cells is observed after IR (ionizing irradiation ) with 9.5 Gy (gray). The myeloablation reveals the presence of the open reticular meshwork of the stromal cells. The stromal cells (in blue) are indicated with black arrows in the right panel. A large macrophage containing phagocytized hematopoietic cells is shown with a red arrow in the right panel. Collected at 22nd day following IR. Hematoxylin and eosin staining. Experimental conditions are indicated in Figure 1.

Tissue barriers constitute multiple interfaces with external and internal environments to sustain electrolyte, immunochemical, thermal, and mechanical balance of animal and human body. The barriers such as mucosa, vasculature, skin, respiratory tract, and connective tissue are comprised of numerous lineages of epithelial, endothelial, lymphatic, reticulo-endothelial, and mesenchymal (e.g., stromal fibroblastic, periendothelial mesenchymal, *etc.*) cells, which are all of different radiosensitivity. To date it is well accepted that the cause of hARS is (i) direct and indirect damage to radio-sensitive lineages in bone marrow, lymphoid tissue, gut mucosa, and microvascular endothelium; (ii) overwhelming reactive responses leading to hypercytokinemia and massive release of toxic products; and (iii) loss of integrity of tissue barriers and systemic homeostatic control [1,2,3,4,5,10,11,13,15,16,20,44,51,59,82,87,90,92]. In the context of these pathophysiological events it is unclear what are the leading cell lineages responsible for: (i) prolonged production of ROS, RNS, RCS; (ii) respective up-regulation of oxidative and electrophilic signaling, and cytotoxicity; and (iii) associated alterations in barrier functions. Let’s say, considering that both severe myeloablation and depletion of lymphoid tissue is a feature of the “latent” period (Figure 1 and Figure 3) one would unlikely expect a substantial contribution of hematopoietic cells, and circulatory leukocytes in the delayed effects of oxidative stress in bone marrow, mucosa and microvasculature, which we have discussed above. However, that balance could be different at prodrome period as well as after hematopoietic recovery (if occurs) [44].

A growing body of data suggests emerging role of “radioresistant” lineages, such as Paneth cells, mural cells, microglia, astrocytes, stromal cells, *etc.*, in reactive pro-inflammatory and cytotoxic effects on progenitor cell in dose limited tissues [49,57,77,91,92,94]. Thus, protracted pro-oxidative effects in irradiated bone marrow, vasculature, brain and intestine were attributed to activation ROS-producing mechanisms in microglia, astrocytes, and mural cells, to up-regulation of NO-producing mechanisms, and to accumulation of lipid-derived carbonyl species in gut crypt cells and stromal cells [43,49,57,77,91,92,94]. These phenomena can cause susceptibility of major tissue barriers to post-radiation remodeling and their loss of homeostatic control [16,20,44,51,92,94,95].

Fibroblastic mesenchymal stromal cells (thereafter stromal cells; MSCs) are components of stroma of all origins that sustains architecture of soft tissues and is attributed to connective tissue [63,64,65]. Although MSCs are considered to be ubiquitously integrated into stroma of vascular, skin, lung, intestinal, adiposal and other tissues, their major source in the body is the bone marrow stroma [17,25,63,64,65]. It should be noted, that MSCs extracted from normal bone marrow *per se* exert numerous remarkable properties, which include but not limited to antiseptic, immunomodulatory, tissue regenerative activity, and ability to sustain mitochondrial homeostasis in damaged tissue barriers by intracellular exchange with functional mitochondrial or regulatory microvesicles [18,29,62,63,64,96]. Overall, these observations suggest that fibroblastic stromal cells can express molecular mechanisms to modulate functional microenvironment in stromal tissue, perivascular network, vascular endothelium, and parenchymal tissue. Interestingly, MSCs are able to promptly transfer to pro-inflammatory phenotypes after irradiation, challenge with bacterial inflammagens, or a combination thereof [61,62,94]. This fact is important for understanding the role of irradiated perivascular stromal cells in response to the radiation-induced septicemia, since bacteria spread in blood and tissue colonization should inevitably impact these vascular components.

MSCs are shown to be relatively resistant to irradiation and remain in bone marrow after IR-induced depletion with hematopoietic cells (Figure 3), [44,57]. The recent *in vitro* data show that the MSC responses to IR with doses 8–12 Gy (^60^Co gamma-photon) and challenge with bacterial inflammagens are characterized by cytostatic effects, suppression of clonogenicity, and activation of numerous redox-adaptive and repair mechanisms sustaining their survival from the photon hit and following metabolic stress [61,62,95]. Thus, while MSCs displayed high levels of constitutively present adaptogens, for example, HSP70 and mitochondrial Sirt3, irradiation and treatments with LPS or *E. coli* can induce a number of adaptive responses that included induction and nuclear translocation of redox response factors such as NFκB, TRX1, Ref1, Nrf2, FoxO3a, expression of NFκB, heme oxygenase 1, MMP3, numerous pro-inflammatory cytokines, mitochondrial remodeling and autophagy of aberrant mitochondria [61,62,96]. The induced MSC changes to pro-inflammatory phenotype can also lead to increase in cell production of reactive species able to oxidize dihydrorhodamine 123 and 4-amino-5-methylamino-2′,7′-difluorofluorescein (DAF-FM) [57,62].

Fibroblastic stromal cells constitute a large network in the body. With regards to microvasculature, the two-step impact developing in hARS, *i.e.*, radiation and sepsis, can cause dramatic reactive responses (including massive oxidative stress to stromal milieu) in MSCs. The possible outcomes may include: (i) impairment of endothelial-mesenchymal interaction in the microvasculature; (ii) endothelial shedding; (iii) increase in vascular permeability; (iv) interstitial hemorrhage; and (v) oxidative stress mediated by extravasated hemoglobin.

## 4. Conclusions

Recent reports indicate that primary radiolytic hit of acute ionizing irradiation induces a wide range of morphological, cytodynamic, pro-inflammatory, and metabolic changes in tissues and systems. These effects include a dramatic impact on cell redox-pathways such as mitochondrial respiration, synthesis of nitric oxide, NADPH oxidase respiratory burst, *etc*. Dysregulation of these pathways can lead to: (i) overwhelming generation of a plethora of reactive species (*i.e.*, ROS, RNS, and reactive products of oxidation of lipids, proteins, and nucleic acids); (ii) the subsequent proteotoxic stress and numerous deleterious targeted and non-targeted genotoxic effects; and (iii) induction of a cascade of adaptive responses in order to sustain the imbalanced proteostasis (*i.e.*, homeostasis of the proteome), genomic homeostasis and the altered lipid and general metabolomic profiles [4,6,7,11,16,18,27,28,35,36,38,42,43,45,66,76,79,83,91,92,94,97,98]. In hARS the line of these events may have a protracted character and contribute to increase in susceptibility of sensitive tissues to a secondary hit caused by bacterial inflammagens appeared in circulation due to delayed bacterial breach of tissue barriers. An array of reactive responses to the septicemia in vasculature and parenchymal tissues can abrupt homeostatic control systems culminating in MOF. Interplay between protracted oxidative stress, the redox metabolome, redox interactome and functional genome that mediate redox homeostasis in tissue barriers requires further clarifications.
